# Inflammation of mammary adipose tissue occurs in overweight and obese patients exhibiting early-stage breast cancer

**DOI:** 10.1038/s41523-017-0015-9

**Published:** 2017-05-03

**Authors:** Charlotte Vaysse, Jon Lømo, Øystein Garred, Frøydis Fjeldheim, Trygve Lofteroed, Ellen Schlichting, Anne McTiernan, Hanne Frydenberg, Anders Husøy, Steinar Lundgren, Morten W. Fagerland, Elin Richardsen, Erik A. Wist, Catherine Muller, Inger Thune

**Affiliations:** 1 The Cancer Center, Oslo University Hospital, Oslo, Norway; 2 Institut de Pharmacologie et de Biologie Structurale (IPBS), Université de Toulouse, CNRS, UPS, Toulouse, France; 3 Department of Pathology, Oslo University Hospital, Oslo, Norway; 4 Institute of Clinical Medicine, University of Oslo, Oslo, Norway; 5 Department of Breast and Endocrine Surgery, Oslo University Hospital, Oslo, Norway; 6 Fred Hutchinson Cancer Research Center, Public Health Sciences Division, Seattle, WA USA; 7 Department of Oncology, St. Olavs University Hospital, Trondheim, Norway; 8 Centre for Biostatistics and Epidemiology, Research Support Services, Oslo University Hospital Oslo, Oslo, Norway; 9 Department of Sports Medicine, Norwegian School of Sport Sciences, Oslo, Norway; 10 Department of Medical Biology, Department of Clinical Pathology, UiT The Arctic University of Norway, University of North Norway, Tromsø, Norway; 11 Institute of Clinical Medicine, Faculty of Health Sciences, University of Tromsø, Tromsø, Norway

## Abstract

Growing evidence indicates that adiposity is associated with breast cancer risk and negatively affects breast cancer recurrence and survival, a paracrine role of mammary adipose tissue being very likely in this process. In contrast to other adipose depots, occurrence of a sub-inflammatory state of mammary adipose tissue defined by dying adipocytes surrounded by macrophages forming crown-like structures in overweight and obese subjects, remains only partially described. In a general population of breast cancer patients (107 patients) mostly undergoing breast-conserving surgery, we found a positive association between patient’s body composition, breast adipocytes size, and presence of crown-like structures in mammary adipose tissue close to the tumor. Overweight (BMI: 25.0–29.9 kg/m^2^) and obese (BMI ≥ 30.0 kg/m^2^) patients have 3.2 and 6.9 times higher odds ratio of crown-like structures respectively, compared with normal weight patients. The relatively small increase in adipocyte size in crown-like structures positive vs. negative patients suggests that mammary adipose tissue inflammation might occur early during hypertrophy. Our results further highlight that body mass index is an adequate predictor of the presence of crown-like structures in mammary adipose tissue among postmenopausal women, whereas in premenopausal women truncal fat percentage might be more predictive, suggesting that mammary adipose tissue inflammation is more likely to occur in patients exhibiting visceral obesity. Finally, the presence of crown-like structures was positively associated with systemic markers such as the Triglyceride/High-density lipoprotein-cholesterol ratio serum C-reactive protein and glucose/(HbA1c) glycated Haemoglobin. These compelling results demonstrate that excess adiposity, even in overweight patients, is associated with mammary adipose tissue inflammation, an event that could contribute to breast cancer development and progression.

## Immunology: Weight tied to inflammation in fat surrounding tumor

Overweight and obese women with breast cancer show more inflammation in their mammary fat tissue, creating an environment favorable to tumor growth. In a study performed at the Oslo University Hospital, Norway, Charlotte Vaysse and colleagues characterized the fat cells found close to the breast tumors of 107 patients with early-stage disease. The researchers showed that overweight and obese women were more likely to have clusters of pro-inflammatory macrophage cells within the fat tissue close to the tumors than normal weight women. They further divided the patients according to whether they’d gone through menopause or not, and found that body mass index was a good predictor of fat cell inflammatory status in postmenopausal women, whereas belly fat percentage was a more accurate measure for premenopausal women. The inflammation brought on by excess weight may contribute to breast cancer development and progression.

## Introduction

Growing evidence from both clinical and preclinical studies indicates that adiposity is associated with breast cancer risk,^1, 2^ and may act as a negative prognostic factor influencing breast cancer recurrence and survival.^1–3^ These observations have recently been supported in mechanistic studies, observing that adiposity-associated factors, such as hormones, lipids, adipokines, and pro-inflammatory mediators are associated with breast cancer development and progression.^4, 5^

Accumulating studies point to a role of mammary adipose tissue (MAT) adjacent to the tumors in breast cancer development and progression, as adipose tissue (AT) represents a major component of the breast tumor microenvironment.^6, 7^ We and others have demonstrated that a bidirectional crosstalk takes place between breast cancer cells and tumor-surrounding AT.^8–13^ Tumor-surrounding adipocytes may stimulate the aggressiveness of cancer cells by secreting extracellular matrix such as collagen VI and its fragment endotrophin,^11, 12^ matrix metalloproteases,^13^ chemokines,^10^ and pro-inflammatory cytokines such as Interleukin-6 (IL6).^14^ IL-6 is an important growth factor for estrogen receptor-α (ERα)-positive breast cancer, and elevated serum IL-6 is associated with poor prognosis.^15, 16^ These results strongly support the innovative concept that adipocytes may participate in a highly complex cycle orchestrated by cancer cells to support tumor initiation, growth, and metastasis, possibly amplified in overweight and obese breast cancer patients.

In obesity, both the cellar composition and the secretion of various bioactive substances of AT is deregulated, generating a low-grade, chronic inflammatory state.^4, 5, 17^ This inflammatory state is due to the recruitment of macrophages.^18^ During weight gain, there is a shift in macrophage sub-type to a pro-inflammatory, M1 polarization,^18, 19^ leading to chronic inflammation of the AT with pro-inflammatory release of TNF-α, IL-6, IL-1β, PAI-1 (reviewed in ref. 18). In a very elegant study (after in vivo labeling of macrophages), Lumeng et al. demonstrated that the obesity-induced switch in macrophages activation state is due to the recruitment of additional macrophages from the circulation with high M1 gene expression, that then forms clusters surrounding necrotic adipocytes.^20^ It is therefore now recognized that these clusters (also called crown-like structure or CLS) represent inflammation foci.^20^ Interestingly, the critical size of adipocyte-triggering death is dependent on the site of the adipose depot, in line with the view that adipocytes found in different depots have different properties.^21^ Independently of a cancer context, it has been shown that obesity-induced macrophage infiltration occurs also in MAT and this was, as expected, associated with an increase in pro-inflammatory gene expression.^22, 23^

Existence of such an inflammatory state in MAT is likely to explain an amplification of its paracrine effect on tumor development and progression among overweight and obese women. In contrast to sub-cutaneous adipose tissue (SAT) and visceral adipose tissue (VAT),^21, 24^ less is known about CLS accumulation in MAT in obese, but also in overweight women. The pioneering work of Dannenberg and collaborators has demonstrated that CLS occurs in the MAT of obese subjects bearing breast cancers.^23, 25^ However, several questions remain unclear concerning the link between CLS accumulation in MAT and overweight/obesity in a cancer context. Previous studies were performed in mastectomy specimens, including a vast majority of patients with aggressive and high-grade tumors and positive lymph nodes involvement.^25–27^ Thus, it remains undetermined if CLS also occurs in localized early-stage breast tumors. Another major issue is whether body mass index (BMI), common clinical measure of body composition, is the most appropriate measure of adiposity to reflect the presence of CLS in MAT, or whether other anthropometric measures focused on abdominal obesity such as waist to hip ratio (WHR) or truncal fat measured with Dual X-ray Absorptiometry scan (DXA) are more appropriate. Finally, the associations among CLS, metabolic dysfunction and low grade inflammation (such as glucose/HbA1c levels, dyslipidemia and level of C-Reactive protein, CRP) have been focused on only in one study^25^ and need to be further investigated.

Thus, the main aim of the present study was to elaborate on whether MAT inflammation in lumpectomy specimens, as reflected by CLS, is associated with mammary adipocyte size, body composition assessed by various methods of assessments, and serum biomarkers, in patients with the most common types of breast tumors in routine clinical practice.

## Results

### Patient characteristics

Breast cancer patients had the following means: 55.2 years age at diagnosis, BMI of 24.9 kg/m^2^, WHR of 0.88, % truncal fat of 37.3%, serum Triglycerides (TG) of 1.07 mmol/L and cholesterol of 5.61 mmmol/L, and a median serum CRP of 0.80 mg/L. Most of the women underwent breast conservative surgery (71.0%) (Table 1). Compared with lean/normal weight patients, overweight/obese patients were slightly older and had higher WHR and % truncal fat (*P* < 0.001). In addition, the overweight and obese patients exhibit unfavorable lipid profiles compared to lean/normal weighted ones, including higher serum total cholesterol (5.88 vs. 5.42 mmol/L, *P* = 0.014), lower serum High-density lipoprotein (HDL)-cholesterol (1.74 vs. 1.98 mmol/L, *P* = 0.026) and higher serum TG (1.36 vs. 0.85 mmol/L, *P < *0.001), as well as significant increase in glucose (5.63 vs. 5.27 mmol/L, *P* = 0.040), but not not necessarily with HBA1c, levels (Table 1).

**Table 1:** Characteristics of the breast cancer patients (hosts) and the breast tumors, overall and stratified by BMI; presented as means (SD) or % (n)

Characteristics	Total	BMI		
	(*n* = 107)^a^	<25 kg/m^2^ (*n* = 62)^a^	≥25 kg/m^2^ (*n* = 45)^a^	*P*-value
	Mean (*SD*)/% (*n*)	Mean (*SD*)/%(*n*)	Mean (*SD*)/%(*n*)	
Age at diagnosis, years	55.2 (8.17)	53.7 (7.8)	57.2 (8.3)	0.025
Number of children	1.59 (1.18)	1.55 (1.18)	1.64 (1.19)	0.680
Postmenopausal	70.1 (75)	62.9 (39)	80.0 (36)	0.057
Body fat measures				
BMI, kg/m^2^	24.9 (3.65)	22.3 (1.8)	28.3 (2.6)	<0.001
WHR, cm	0.88 (0.06)	0.86 (0.05)	0.90 (0.05)	<0.001
DXA, fat trunk percent	37.3 (9.47)	32.4 (8.2)	44.0 (6.6)	<0.001
Serum biomarkers				
Cholesterol, mmol/L	5.61 (0.96)	5.42 (0.91)	5.88 (0.96)	0.014
HDL-cholesterol, mmol/L	1.88 (0.55)	1.98 (0.49)	1.74 (0.59)	0.026
LDL-cholesterol, mmol/L	3.38 (0.91)	3.18 (0.86)	3.66 (0.90)	0.007
Triglycerides, mmol/L	1.07 (0.53)	0.85 (0.35)	1.36 (0.60)	<0.001
CRP, mg/L^b^	0.80 (2.10)	0.00 (1.23)	1.80 (2.60)	<0.001
Glucose, mmol/L	5.43 (0.64)	5.27 (0.61)	5.63 (0.62)	0.004
HbA1c	5.48 (0.38)	5.43 (0.41)	5.56 (0.34)	0.103
Medications				
Hormone users, %	29.9 (32)	32.3 (20)	26.7 (12)	0.530
Statin users, %	4.67 (5)	4.84 (3)	4.44 (2)	0.820
NSAID users, %	41.1 (44)	40.3 (25)	42.2 (19)	0.840
Surgical treatment				
Conservative surgery, %	71.0 (76)	59.7 (37)	86.7 (39)	0.003
Mastectomy, %	29.0 (31)	40.3 (25)	13.3 (6)	
Right breast, %	43.0(46)	38.7 (24)	48.9 (22)	0.111
Bilateral, %	1.90 (2)	0	4.44 (2)	
Breast tumor characteristics				
Histology				
Invasive ductal carcinoma, %	81.1(86)	80.3 (49)	82.2 (37)	
Invasive lobular carcinoma, %	13.2 (14)	14.8 (9)	11.1 (5)	0.814
Others, %	5.61 (6)	4.84 (3)	6.67 (3)	
Tumor diameter, mm	16.4 (9.55)	17.3 (10.7)	15.2 (7.50)	0.265
Grade, %				
1	30.8 (33)	27.4 (17)	35.6 (16)	
2	47.7 (51)	48.4 (30)	46.7 (21)	0.584
3	21.5 (23)	24.2 (15)	17.8 (8)	
Ki67 hotspot, %	29.4 (21.6)	30.9 (22.0)	27.2 (21.0)	0.388
Nodal metastasis positive, %	25.2 (27)	27.4 (17)	22.2 (10)	0.654
ER positive, %	90.7 (97)	90.3 (56)	91.1 (41)	1.000
ER percent	84.2 (30.4)	82.9 (30.8)	86.0 (30.1)	0.607
PgR positive, %	69.2 (74)	72.6 (45)	64.6 (29)	0.402
PgR percent	52.4 (41.1)	53.1 (40.0)	51.4 (43.0)	0.842
HER2 positive, %	5.61 (6)	6.45 (4)	4.44 (2)	1.000
Fat tissue surrounding tumor				
CLS density, CLS/cm^2 b^	0.30 (1.30)	0.00 (0.66)	1.03 (2.00)	<0.001
Area fat tissue, cm^2^	2.01 (1.06)	1.85 (0.93)	2.23 (1.20)	0.076
Adipocyte diameter, µm	64.3 (12.9)	60.4 (11.9)	69.7 (12.4)	<0.001
Adipocyte number, n	306 (120)	341 (130)	257 (85.3)	<0.001

*SD* standard deviation, *BMI* body mass index (kg/m^2^), *WHR* waist-hip ratio, *HDL* high-density lipoprotein, *LDL*, low-density lipoprotein, *CRP* C-reactive protein, *NSAID* nonsteroidal anti-inflammatory drugs, *ER* estrogen receptor (ER positive when ≥1%), *PgR* progesterone receptor (PgR positive when ≥10%), *HER2* human epidermal growth factor receptor 2, *CLS* crown-like structure

^a^ Number of patients may vary due to missing information

^b^ Median and interquartile range

### Characteristics of the breast tumor and fat tissue surrounding tumor

**Fig. 1:**
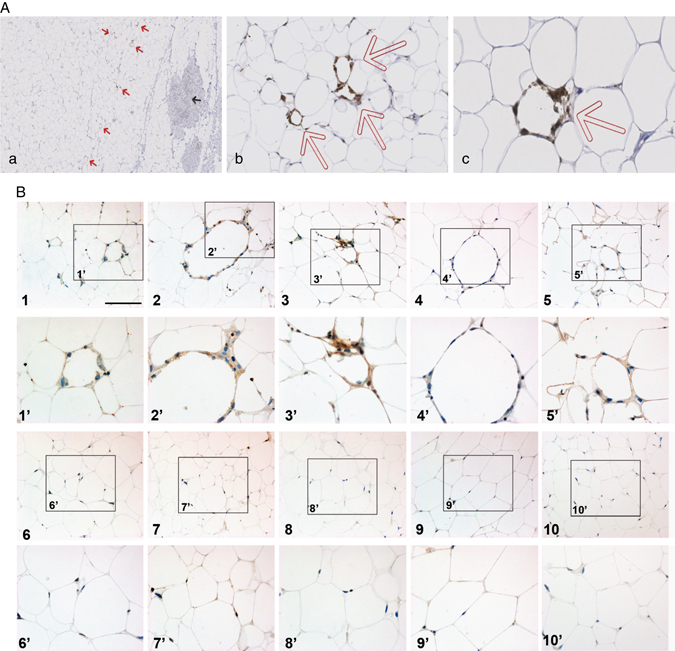
CLS are found in MAT and macrophages constituting CLS are pro-inflammatory. **a** Photomicrographs of CD68 stained tumor slides showing macrophages in isolated ring-like formations surrounding dying or dead adipocytes (indicated by *red arrows*). Density of CLS in breast fat tissue away from tumor border was scored as number of CLS divided by area of fat tissue. **a** ×25 magnification, *red arrow*: CLS, *black arrow*: tumor; **b** ×150 magnification; **c** ×300 magnification of CLS detected in the MAT of an obese patient (BMI = 30 kg/m^2^) exhibiting hypertrophied adipocyte (mean adipocyte diameter 82.09 µm). **b** IL6 expression was evaluated in 5 samples exhibiting CLS obtained from overweight patients (BMI from 26.4 to 29.5 kg/m^2^) (Samples 1 to 5) (×200 magnification). The figures below (numbered 1’ to 5’) show two-fold-magnified views of selected areas indicated by insets. Similar experiments were performed in samples without CLS obtained from normal weight patients (BMI from 17.9 to 24.8 kg/m^2^) numbered (6 to 10), with two fold-magnified views of selected areas (6’ to 10’)

Breast tumors had a mean size of 16.4 mm. The majority of tumors were invasive ductal carcinomas (81.1%) and ERα-positive (90.7%). Only 25.2% of the patients had nodal metastases. A representative picture of CLS showing CD68 positive macrophages in ring-like formation surrounding dying or dead adipocytes is presented in Fig. 1a. As shown in Fig. 1b, macrophages constituting CLS were positive for IL6 expression, highlighting their pro-inflammatory status. The median CLS density was 0.30 CLS/cm^2^ for a mean area of AT of 2.01 cm^2^. The mean size of adipocytes was 64.3 μm with a mean count of 306 adipocytes per slide (a representative figure of the method used is shown in Supplementary Figure 1).

### Adipocyte size, CLS, and anthropometric measures

**Fig. 2:**
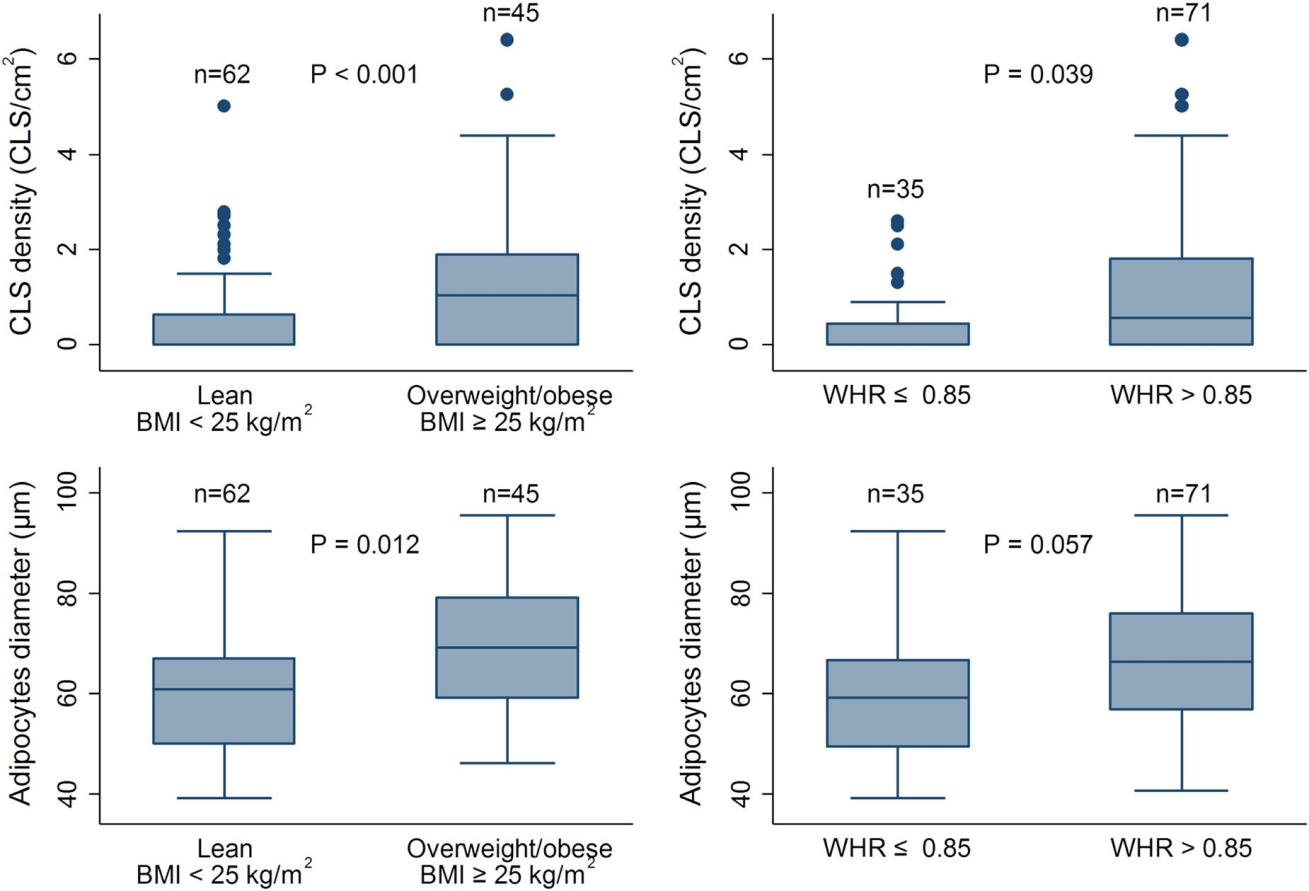
Distribution of CLS density (*top panel*) or adipocyte diameter (*bottom panel*), stratified in lean vs. overweight/obese patients, as measured either by BMI (*left panel*) or WHR (*right panel*). The *P*-values are obtained from tests of equal medians

We observed a higher CLS density among overweight/obese patients compared to the lean/normal weight patients (*P* < 0.001, Table 1, Fig. 2). The mean adipocyte size was also higher in overweight/obese patients (mean 69.7 µm) compared to lean patients (60.4 µm, *P* = 0.012). Similar results were obtained when patients were split by WHR categories (Fig. 2).

**Fig. 3:**
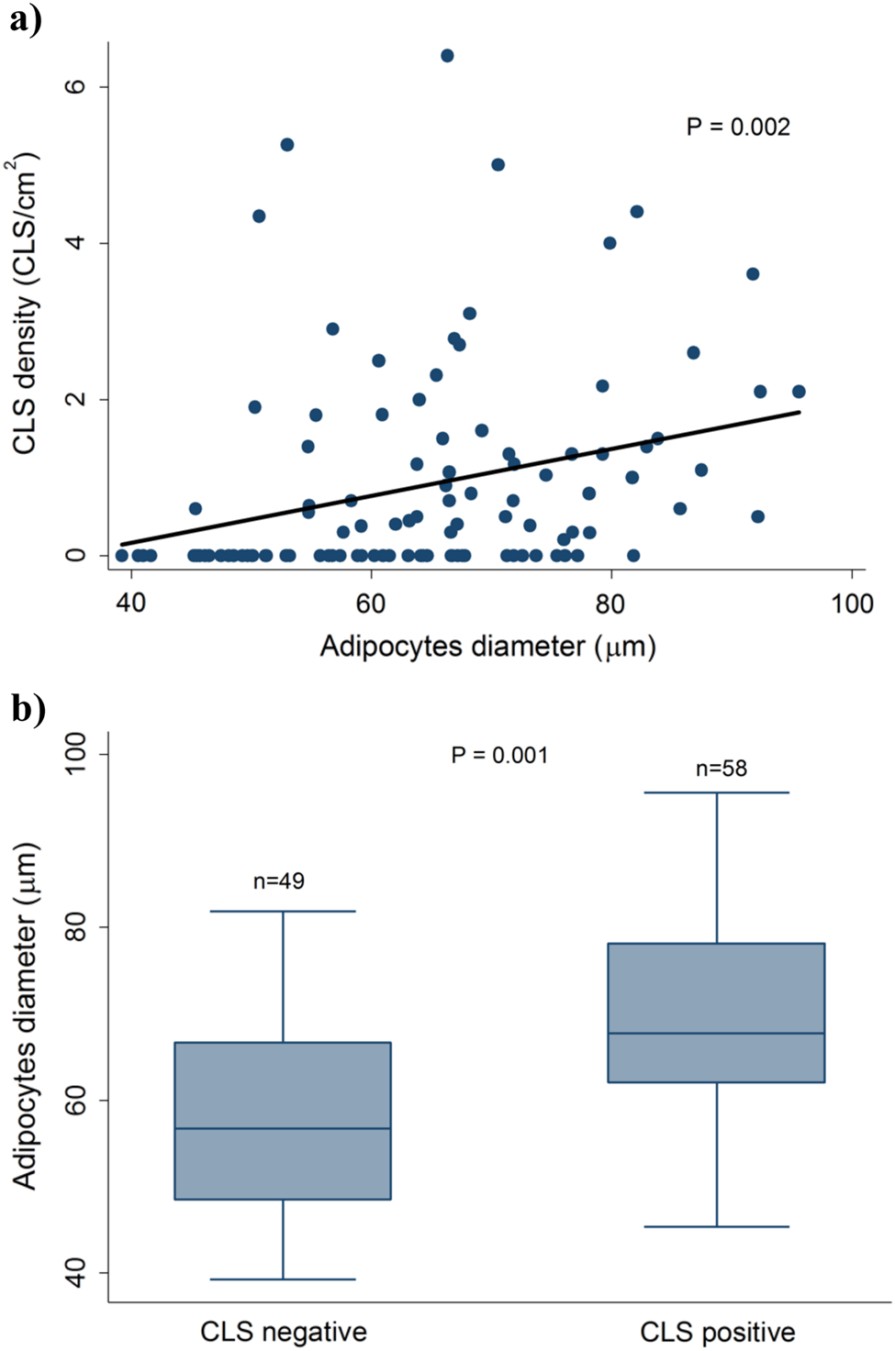
Existence of a relationship between CLS density and adipocyte size. **a** Scatter plot and linear relationship of CLS density and adipocyte diameter. The *P*-value is obtained from a test of no linear association. **b** Box plot that shows the distribution of adipocytes diameter, stratified by presence of CLS

We observed a positive linear relationship between adipocyte size and CLS density, but no threshold effect was identified (Fig. 3a). When we split the patients in two categories according to CLS presence a higher adipocyte diameter was observed among the CLS positive patients compared to the CLS negative patients (*P* = 0.001) (Fig. 3b).

**Fig. 4:**
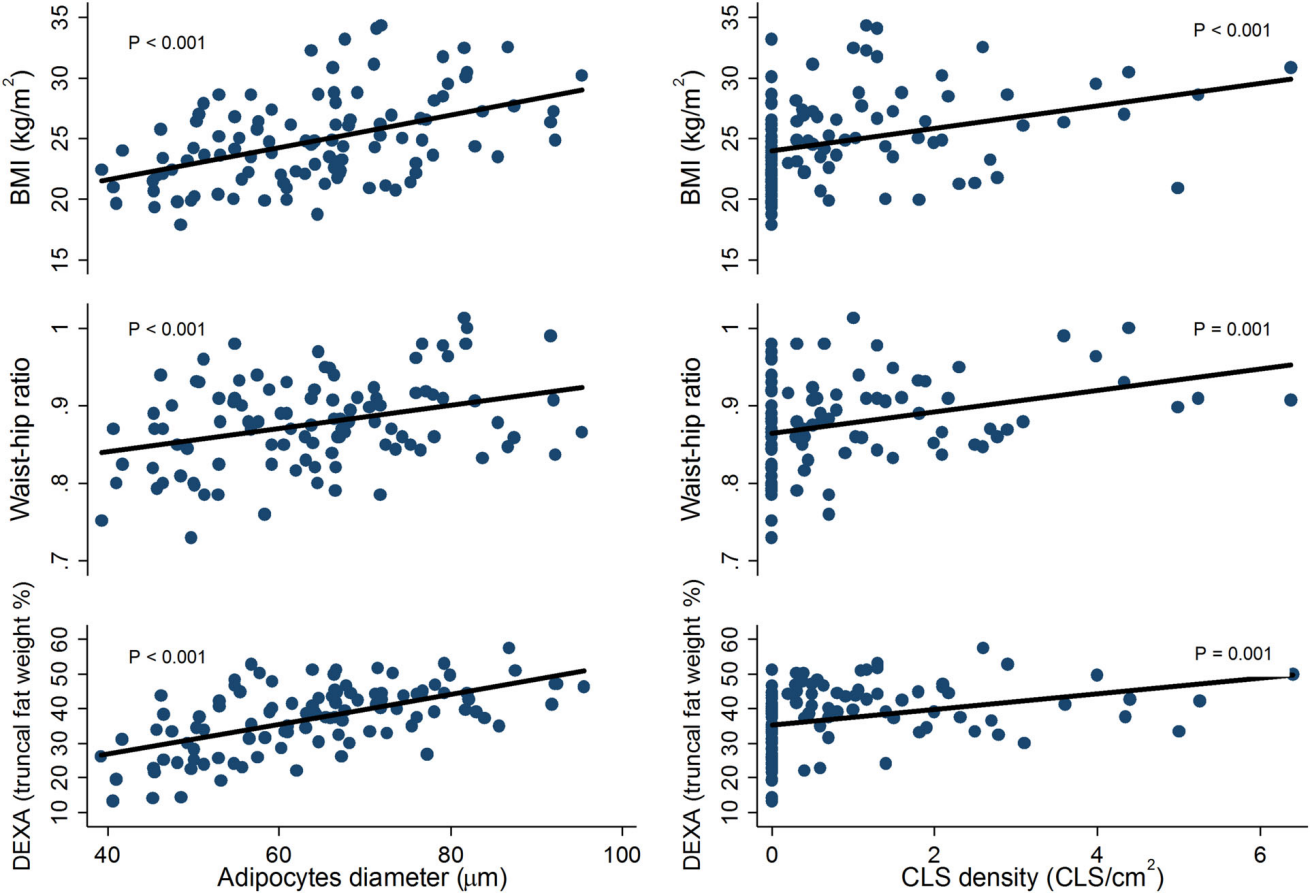
Scatterplots and linear relationships of anthropometric measures (*y*-axis) and adipocyte diameter (*x*-axis, *left panel*) or CLS density (*x*-axis, *right panel*). The *P*-values are obtained from tests of no linear association

We then investigated the associations between the different anthropometric measures and MAT dysfunction (adipocyte’s hypertrophy and CLS density). We observed a positive linear association without threshold effects between anthropometric measures (BMI, WHR, % truncal fat) and breast adipocytes size (µm) (*P* < 0.001), and between anthropometric measures and CLS density (CLS/cm^2^); BMI (*P* < 0.001), WHR (*P* = 0.001), and % truncal fat (*P* = 0.001) (Fig. 4). We further split the patients by menopausal status, and the same associations were observed (Supplementary Figure 2).

Using logistic regression, we observed significantly higher odds ratio (OR) of presence of CLS for each kg/m^2^ (Table 2). In categorical analyses, the overweight and obese breast cancer patients had a 3.2 higher OR (95%CI 1.28–8.15), and a 6.9 higher OR (95%CI 1.35–35.0) of CLS, respectively, compared with lean/normal patients.

**Table 2:** Multivariable adjusted odds ratio (OR) for presence of CLS in fat tissue surrounding tumor according to anthropometric and metabolic measures, overall and by menopausal status

Explanatory variables	Overall OR (95% CI*) n* = 107 ^a^	Premenopausal OR (95% CI) *n* = 32^a^	Postmenopausal OR (95% CI) *n* = 75^a^
BMI (kg/m^2^)			
1 kg/m^2^	1.28 (1.11–1.46)	1.30 (0.99–1.70)	1.26 (1.08–1.48)
2 kg/m^2^	1.63 (1.24–2.14)	1.69 (0.99–2.90)	1.59 (1.16–2.19)
5 kg/m^2^	3.39 (1.71–6.73)	3.74 (0.97–14.3)	3.21 (1.45–7.12)
DXA truncal fat (%)			
5 % points	1.96 (1.44–2.66)	2.84 (1.35–5.95)	1.75 (1.25–2.45)
10 % points	3.83 (2.07–7.10)	8.05 (1.83–35.4)	3.05 (1.55–5.98)
1 SD (9.5%)	3.58 (2.00–6.44)	7.26 (1.78–29.6)	2.88 (1.52–5.47)
Waist-hip ratio			
>0.85^b^ vs. ≤0.85^c^	3.26 (1.35–7.85)	2.37 (0.43–13.0)	3.38 (1.19–9.59)
Serum markers			
Cholesterol (1 SD 0.96 mmol/L)	0.95 (0.63–1.44)	1.32 (0.55–3.16)	0.82 (0.50–1.34)
HDL-cholesterol (1 SD, 0.55 mmol/L)	0.76 (0.51–1.13)	0.61 (0.22–1.70)	0.73 (0.45–1.17)
LDL-cholesterol (1 SD, 0.91 mmol/L)	0.92 (0.61–1.39)	1.30 (0.50–3.35)	0.82 (0.52–1.31)
Triglycerides (1 SD, 0.53 mmol/L)	2.00 (1.16–3.42)	0.36 (0.12–1.10)	2.31 (1.13–4.72)
HDL/total cholesterol ratio: (1 SD 0.11)	0.75 (0.49–1.13)	1.86 (0.85–4.08)	0.77 (0.47–1.26)
Triglycerides/HDL-cholesterol (1 SD, 0.52)	1.75 (1.04–2.93)	1.73 (0.84–3.57)	1.97 (0.99–3.91)
Glucose (1 SD, 0.64 mmol/L)	1.94 (1.19–3.16)	1.70 (0.70–4.15)	2.15 (1.17–3.96)
HbA1c (1 SD, 0.38%)	2.23 (1.36–3.67)	7.96 (1.55–40.9)	1.75 (1.03–2.98)
CRP (1 interquartile range (2.1 mg/L)	1.10 (0.82–1.48)	7.05 (1.39–35.8)	0.99 (0.75–1.31)

Logistic regression model

Numbers may vary due to missing information

*BMI* body mass index (kg/m^2^), *CI* confidence interval, *CLS* crown like structures, *HDL* high-density lipoprotein, *CRP* C-reactive protein, *LDL* low-density lipoprotein, *n* cases, *SD* standard deviation, *vs* versus

^a^ Adjusted for age, parity, and hormone replacement therapy (HRT) use

^b^ Obese

^c^ Normal/overweight

Compared to women with WHR ≤ 0.85, those with WHR > 0.85 had an OR of 3.26 (95% CI 1.35–7.85) of presence of CLS; this effect was observed in both premenopausal and postmenopausal women (Table 2). When we studied the association between % truncal fat and the presence of CLS, we observed by each 10 % higher truncal fat, a 3.83 higher OR (95% CI 2.07–7.10) of CLS presence in the overall population. The OR was consistently higher in premenopausal than in postmenopausal women (Table 2).

### Serum lipids and inflammatory systemic markers

We observed a 2.00 higher OR, (95% CI 1.16–3.42) for presence of CLS for each standard deviation (SD) increase in TG (Table 2). A similar increase in CLS was seen for TG in postmenopausal, but not premenopausal, breast cancer patients. Among the postmenopausal patients, an increase of 0.53 mmol/L (SD) in TG was associated with 2.3 times higher odds of CLS presence (OR 2.32, 95% CI 1.13–4.72). An increase in TG/HDL-cholesterol ratio by 0.52 (SD) was associated with an overall OR of 1.75 (95% CI 1.04–2.93) of presence of CLS, and this association was seen in both postmenopausal and premenopausal women. An increased CRP (1 interquartile range, 2.1) was associated with 7.1 times higher odds of CLS presence (OR 7.05, 95% CI 1.39–35.8) in premenopausal women only (Table 2); no such higher odds were seen in postmenopausal women. An increase of 0.64 mmol/L (SD) in glucose was associated with 1.94 times higher odds of CLS presence (OR 1.94, 95% CI 1.19–3.16), and almost similar results were seen in both postmenopausal and premenopausal women. Increase in HbA1C levels (1 SD, 0.38 %), was associated with a 2.23 higher odds of CLS presence in the overall population, but a higher odds of CLS presence could be seen in premenopausal (7.96) than in postmenopausal women (1.75) (Table 2). No significant associations were observed between higher total cholesterol, HDL-cholesterol, Low-density lipoprotein (LDL)-cholesterol, or HDL-cholesterol/total-cholesterol ratio, and CLS presence. Finally, when we compared the metabolic parameters of CLS positive patients (*n* = 58) to those of CLS negative patients, TG, TG/HDL-cholesterol as well as glucose and HbA1c levels were higher in CLS positive than in CLS negative patients (Supplementary Table 1).

## Discussion

In this sample of breast cancer patients, 71% of whom underwent breast-conserving surgery, we observed a strong positive correlation between patient’s body composition, breast adipocytes size, and the presence of CLS in MAT close to the tumor, likely reflecting local inflammation. Our results further highlight that BMI is an adequate predictor of the presence of CLS in MAT among postmenopausal women, whereas the measure of truncal fat percentage might be more predictive in premenopausal women. Our study also underlines that CLS are present in overweight patients in accordance with the fact that a relatively small increase in adipocyte size is observed between CLS positive and negative patients, suggesting that this tissue might be prone to inflammation during hypertrophy. Finally, the presence of CLS was associated with systemic markers such as TG/HDL-cholesterol ratio, TG (in the postmenopausal population), glucose/HbA1C whereas serum CRP was associated with the presence of CLS in premenopausal women. These compelling results demonstrate that excess adiposity is associated with MAT inflammation, a condition that could contribute to breast cancer development and progression in a paracrine manner.

Our results extend previous observations suggesting that MAT inflammation occurs with excess adiposity as previously observed for others fat depots such as SAT and VAT. In fact, only a limited number of studies have reported the presence of CLS in MAT of overweight/obese patients with breast cancers, and all emanated from the same research group.^26–28^ Our study extends previous results, as we also demonstrate that the presence of CLS occurs in a series of overweight/obese patients that mostly exhibited low-grade tumors treated by breast tumor resection. This is in contrast to previous studies that were performed on mastectomy specimens including a majority of patients with aggressive, high grade tumors and positive lymph node involvement.^26–28^ Importantly, in our study, the analyses of CLS were performed in fat tissue in the same block as the tumors, and therefore were in close proximity to the tumor mass. Moreover, by including both breast conservative and radical surgeries, our study reflects the current clinical practices on management of breast cancer stage I and II with the evolution of techniques and therapeutics.^29^ Our findings therefore highlight that MAT inflammation associated with excess weight might have important implications in clinical practice since it concerns a large majority of patients treated for breast cancer.

All three methods of measuring body composition in this study showed a significant correlation between CLS density and adipocyte size in breast tissue (Fig. 4). Importantly, this correlation was found for BMI, which is considered a rough measurement of body composition but is still commonly used in clinical practice.^1, 30^ WHR and truncal fat percentage^31^ were also strongly correlated with adipocyte size and CLS density. When OR were calculated, all the three markers BMI, WHR and truncal fat percentage assessed by DXA predict the occurrence of CLS in the overall population. However, while BMI predicted presence of CLS in postmenopausal women, truncal fat was a better predictor in premenopausal women. After menopause, increase in BMI is associated with increased amounts of visceral adiposity (to the same levels of male subjects) whereas a lower incidence of visceral obesity according to BMI is observed in premenopausal women.^32^ Accordingly, after menopause the BMI reflects abdominal distribution of fat whereas in premenopausal women, these two variables could be dissociated.^32^ In obesity, VAT is more inflammatory than SAT and the accumulation of VAT is strongly associated with obesity-related complications like Type 2 diabetes and coronary artery disease.^33^ Interestingly, a higher odds of CLS presence for HbA1c levels (that indirectly reflect insulin resistance) was seen in premenopausal (7.96) than in postmenopausal women (1.75). Therefore, our results suggest that MAT inflammation is more likely to occur in patients exhibiting visceral adiposity (and potentially a metabolic syndrome). This link deserves further investigations since it could have important consequences at clinical levels.

Our study shows that the occurrence of CLS in MAT is not limited to obese patients since overweight patients had a 3-fold higher risk of CLS presence compared with lean/normal-weight patients. These results indicate that MAT inflammation might occur even with moderate excess of AT. In regression analysis, a linear association between size of mammary adipocytes and CLS-density was observed suggesting that when breast adipocytes reach a critical size they die, triggering macrophage infiltration, as observed also in AT in other locations.^21, 34, 35^ Interestingly, the increase in adipocyte size in samples that contained CLS was rather modest (1.08-fold, mean size 69.7 µm in CLS positive vs. 64.3 μm in CLS negative samples) in accordance with a previous study also performed in MAT.^36^ These results are in contrast with the 2-fold increase previously reported in both SAT and VAT.^34^ Taken together, these results suggest that the adipocyte size limit triggering cell death in MAT may be smaller than the one observed in other adipose depots, therefore explaining the occurrence of CLS in overweight patients found in our study. Additional studies directly measuring the number of dead adipocytes in AT (as determined by the rate of adipocyte death and the rate of dead adipocyte clearance by macrophages) are needed to confirm this hypothesis.

It is now clearly established that the presence of CLS is associated with a pro-inflammatory response in the affected tissue.^21, 24^ In fact, we demonstrated here that macrophages forming CLS were positive for IL6 expression in all the studied samples. This pro-inflammatory environment is likely to favor the progression of estrogen receptor (ER) + positive samples that represents the vast majority of our samples. Indeed, several of the proinflammatory mediators associated with CLS, including TNFα, IL-1β, IL-6, and Prostaglandin E2 (PGE_2_) are known to up regulate aromatase expression (reviewed in^36^). Very interestingly, it has been demonstrated that cyclooxygenase (COX)-2-derived PGE(2) stimulates a transduction pathway contributing to enhance expression of aromatase (the rate-limiting enzyme for estrogen biosynthesis), and elevated progesterone receptor (PgR) levels in breast tissues from overweight and obese women.^28^ This deciphered pathway may contribute to initiation, progression as well as resistance to anti-aromatase therapies of ER + breast cancers in overweight and obese patients.

Inflammation of AT is a key component of the occurrence of insulin resistance and the metabolic syndrome. In our study, an association between systemic markers (CRP, TG/HDL cholesterol ratio, glucose, and HbA1c levels) and the presence of CLS was observed (see Table 2). These systemic markers could also contribute in addition to local inflammation to cancer progression. For example, elevated CRP levels are associated with shortened disease-specific and overall survival in breast cancer patients.^5, 37–39^ Similarly, insulin resistance could contribute directly (through elevated insulin levels) or indirectly (through its effect on the bioavailability of insulin-like growth factor I) to breast cancer occurrence and progression.^40^ Finally, TG/HDL-cholesterol ratio, that may function as a surrogate for insulin resistance,^41^ is also associated with breast cancer occurrence^42^ and prognosis.^43^ Given the increase in unfavorable metabolic profiles worldwide, and their observed negative effects on breast cancer development and prognosis, there is a need of further studies to improve our knowledge regarding the association between components of metabolic syndrome and MAT inflammation.

In conclusion, our findings support that the presence of CLS in MAT is increased with overweight and obesity. Our findings were obtained in a series of patients representing those commonly seen in clinical practice. In addition, we demonstrate that BMI is an adequate measure to predict the presence of CLS in MAT among postmenopausal women, whereas in premenopausal women the measure of truncal fat percentage might be more predictive, highlighting a potential link between visceral adiposity and the presence of CLS in MAT. The presence of CLS in MAT close to the tumors would contribute to generate a pro-inflammatory environment favorable to breast cancer occurrence and progression. In addition to the local modification of AT, changes in metabolic parameters may also contribute to this deleterious environment. Interestingly, two very recent studies, although performed in small number of patients, suggest that the presence of CLS might be associated to breast cancer prognosis with a decrease in progression-free survival^44^ and distant recurrence-free survival.^25^

Patients included in this study are part of a clinical protocol (with a final enrollment of 600 patients) and we thus expect to evaluate the impact of CLS presence in MAT on response to treatment, relapse, survival, with regards to different cancer sub-types. In fact, it will be highly interesting to investigate the occurrence of CLS in sub-groups (i.e., HER2 over-expressing as well as triple negative compared to ER + tumors) according to overweight/obesity that was not possible in the current study due to the low numbers of non-ER + patients. This large prospective longitudinal study will comprehensively investigate the prognostic role of MAT inflammation with its associated circulating abnormalities. If studies confirm the link between CLS and breast cancer prognosis, this could have therapeutic impact.

Several strategies have been developed to target inflammation in cancer patients including for example the use of anti-inflammatory drugs (COX2 inhibitors, aspirin, and anti-inflammatory steroids) or anti-cytokines drugs (anti-IL-6 and anti-TNF-α).^45^ It will therefore be important to design specific clinical trials to evaluate the interest of such anti-inflammatory strategies in the sub-population of CLS positive patients. Additional and larger studies are clearly needed to make these findings of importance in daily clinical practice.

## Materials and methods

### Study design

A total of 107 women aged 25–75 years, diagnosed with histological verified invasive breast cancers stages I-II, were included in a clinical breast cancer study (Energy Balance and Breast Cancer Aspects-II) at the Cancer Center, Oslo University Hospital (OUS), St. Olavs Hospital, Trondheim and Vestre Viken HF, Drammen. Women with known severe illnesses (e.g., heart disease and diabetes) were not included in the present study. All participants signed an informed consent form. The study was approved by the Norwegian Regional Committee for Medical Research Ethics.

### Assessments of clinical variables, body composition, and serum markers

Trained personnel at the research departments of the University hospitals assessed baseline patient characteristics, fasting blood samples, and measurements before treatment (surgery, radiation, chemotherapy). Anthropometric measurements (height, weight, waist and hip circumferences) were performed with patients wearing light clothing and no footwear. Height was measured to the nearest 0.5 cm, and weight to the nearest 0.1 kg on an electronic scale, and BMI (kg/m^2^) was calculated. Waist circumference (cm) was measured in a horizontal line, 2.5 cm above the umbilicus. Hip circumference (cm) was measured at the maximum circumference around the buttocks. DXA measurements were performed with a total body scanner (GE Lunar, Madison, WI, using Prodigy enCORE Version 14, 10,022, software) with subjects wearing only cotton briefs with empty bladders. The trunk included the neck, chest, abdominal, and pelvic areas, with its upper perimeter the inferior edge of the chin, and lower borders intersect the femoral necks without touching the brim of the pelvis.^46^ Truncal fat and lean mass, percentage (%) of fat, and fat distribution were assessed.

Blood samples were drawn after overnight fasting.^47^ Total cholesterol, HDL-cholesterol, TG, CRP, glucose and HbA1c were measured in fresh sera at the Department of Clinical Chemistry, OUS, Ullevål (Roche Diagnostics/ Cobas Integra 800-Cobas 8000, Mannheim, Germany, www.roche.com). Total cholesterol was determined enzymatically using cholesterol esterase and cholesterol oxidase; the intra-assay coefficient of variance (CV) was 6% and the inter-assay CV was 3%. HDL-cholesterol was quantified by a direct assay using polyethylene glycol modified enzymes and dextran sulphate. HDL-cholesterol’s intra-assay CV was 7%, and the inter-assay CV was 4%. Serum TG were assayed by enzymatic hydrolysis with lipase, and had an intra-assay CV of 21%, and inter-assay CV of 4%. LDL-cholesterol was calculated using Friedewalds formula. CRP (mg/L) was measured in fresh sera at the Department of Clinical Chemistry, OUS, Ullevål, Norway. It was assessed by a particle-enhanced immunoturbidimetric assay (Cobas 8000 c 702, Roche Diagnostics, Mannheim, Germany) with reagents from the manufacturer. The detection limit was 0.6 mg/L and the CVs ranged from 8% (for CRP > 3 mg/L) to 15% (for CRP > 3 mg/L).

### Tumor characteristics

All breast cancer tumors were histologically examined and classified according to the invasive histological type (ductal, lobular, others), histological grade (1–3), and tumor diameter (both macroscopically and microscopically, mm). Axillary lymph nodes were investigated to detect macro-metastasis or micro-metastasis, using sentinel lymph node technique or axillary lymphadenectomy.

Tumors were routinely investigated with immunohistochemistry for selected markers: ER, PgR, human epidermal growth factor receptor 2 (HER2), and tumor cell proliferation index (Ki67). ER positive status was defined as ≥1% ER-expressing tumor cells, and PgR positive status as ≥10% PgR-expressing tumor cells. Tumors were investigated with HER2 Dual SISH in situ hybridization kit and the percentage of expression of Ki67 positive tumor cells was determined according to national and international guidelines.^48^ The following antibodies were used: ER (clone SP1), PgR (clone 1E2), HER2 (Pathway anti-HER 2 kit, clone 4B5), and Ki67 (MIB1 antibody), all from Ventana, Roche Diagnostics (Oslo, Norway), except MIB1, which was provided by Dako (Oslo, Norway). Primary antibodies were visualized with Ultraview detection kit from Roche. ER, PgR, and HER2 expression were measured according to the international guidelines.^49^ Hormone receptor expression was given as the average percentage of positive cells in the tumor.

### Adipocyte size and crown-like structures

For all cases, we used the part of the tumor paraffin block that contained the highest amount of surrounding MAT. After hematoxylin eosin staining, MAT away from the tumor border was chosen for quantification of adipocyte size. Two independent observers (JL, CV) blinded to the clinical outcome, performed all scoring. A representative area was photographed at ×100 magnification and a picture file stored. The mean diameter (μm) of adipocytes and number of cells in the picture (approximately 300 cells) were calculated using “Adiposoft” software (version 1.13, source: http://fiji.sc/Adiposoft), a plugin of ImageJ, according to Galarraga’s methodology (Supplementary Figure 1).^50^ Immunohistochemistry for CD68 was carried out on parallel sections from paraffin-embedded tumor blocks on a Ventana BenchMark automated staining platform, using the mouse KP1 antibody, at a 1:3000 dilution (obtained from DAKO, Norway). Primary antibodies were visualized with Ultraview detection kit from Roche. Each slide was scanned (NanoZoomer 2.0-RS, Hamamatsu) and the number of CLS in each section was counted and recorded. CLS were counted in the AT excluding the tumor-fat border, which often contained a general inflammatory reaction. The CLS density was calculated as the number of CLS per square centimeter of AT in the tumor block (CLS/cm^2^). The total area of AT was measured using the software of the scanner (NDP 2.4 version), excluding areas of fibrosis and epithelial structures. To evaluate the CLS count, both Light microscopy (Leica, DMLB) and slides scanned were used. For the detection of IL6 expression in CLS, the rabbit polyclonal anti-IL6 antibody was used at a 1:200 dilution (ab6672, obtained from Abcam, Cambridge, UK). Signal was visualized with Discovery ChromoMap DAB detection kit from Roche.

### Statistical methods

Descriptive characteristics are presented as means (SD) or percentages (n). As BMI (kg/m^2^) is the most frequent anthropometric tool used in clinical practice, the cohort was split into two groups of BMI: lean/normal (BMI < 25 kg/m^2^) and overweight/obese (BMI ≥ 25 kg/m^2^).^51^ Differences in the distribution of characteristics at diagnosis between these two subpopulations were calculated using *t*-tests for continuous variables and chi-squared tests for categorical data.

All variables, except CRP and CLS density were approximately normally distributed. CRP and CLS density were somewhat skewed. Thus, we used median regression to compare the medians of CRP (mg/L) and CLS density (CLS/cm^2^). We used linear regression models to study the associations between anthropometric measures (BMI, WHR, and DXA) and adipocyte diameter and between anthropometric measures and CLS density. To study this association in detail, we categorized patients into WHO definitions of lean/normal weight and overweight/obese according to BMI, and into overweight/lean and obese according to WHR (≤0.85 and >0.85).^51^ We also categorized the patients into those whose tumors were CLS positive vs. CLS negative. We used boxplots to show the distribution of CLS density or adipocyte diameter in lean/normal weight vs. overweight/obese, and the distribution of adipocyte diameter in CLS negative vs. CLS positive.

Several variables were assessed as potential confounders: age (continuous), menopausal status (premenopausal/postmenopausal), statin or nonsteroidal anti-inflammatory drugs treatment (current/past/never), lipid profile (continuous), and tumor characteristics (categorical). However, none of these variables influenced the results and were not included in the final linear regression model. We used multivariable logistic regression models to study the association between various anthropometric factors (BMI, WHR, truncal fat %), and selected serum markers and presence of CLS. Based on potential biological mechanisms influencing CLS presence, we adjusted for age (continuous), parity (continuous), and HRT users (current/past/never).

A logistic regression analysis with presence of CLS as variable response within the overall sample and by menopausal status was performed to evaluate OR for variables of interest, including categories of BMI increase (1, 3, and 5 kg/m^2^) WHR (≤0.85 and >0.85), and categories of truncal fat increase (5, 10%, and 1 SD). The statistical analyses were performed with Stata 14.1 (StataCorp LP, College Station, TX, USA). All *p*-values are two-tailed and considered statistically significant if *P* < 0.05.

## Electronic supplementary material

Supplementary Table 1

Supplementary Figure 1

Supplementary Figure 2
