# Erratum: Inflammation of mammary adipose tissue occurs in overweight and obese patients exhibiting early-stage breast cancer

**DOI:** 10.1038/s41523-017-0030-x

**Published:** 2017-09-05

**Authors:** Charlotte Vaysse, Jon Lømo, Øystein Garred, Frøydis Fjeldheim, Trygve Lofteroed, Ellen Schlichting, Anne McTiernan, Hanne Frydenberg, Anders Husøy, Steinar Lundgren, Morten W. Fagerland, Elin Richardsen, Erik A. Wist, Catherine Muller, Inger Thune

**Affiliations:** 1 The Cancer Centre, Oslo University Hospital, Oslo, Norway; 2 Institut de Pharmacologie et de Biologie Structurale (IPBS), Université de Toulouse, CNRS, UPS, Toulouse, France; 3 Department of Pathology, Oslo University Hospital, Oslo, Norway; 4 Institute of Clinical Medicine, University of Oslo, Oslo, Norway; 5 Department of Breast and Endocrine Surgery, Oslo University Hospital, Oslo, Norway; 6 Fred Hutchinson Cancer Research Center, Public Health Sciences Division, Seattle, WA USA; 7 Department of Oncology, St. Olavs University Hospital, Trondheim, Norway; 8 Centre for Biostatistics and Epidemiology, Research Support Services, Oslo University Hospital Oslo, Oslo, Norway; 9 Department of Sports Medicine, Norwegian School of Sport Sciences, Oslo, Norway; 10 Department of Medical Biology, Department of Clinical Pathology, UiT The Arctic University of Norway, University of North Norway, Tromsø, Norway; 11 Institute of Clinical Medicine, Faculty of Health Sciences, University of Tromsø, Tromsø, Norway

**Erratum to:** *npj Breast Cancer* (2017); doi:10.1038/s41523-017-0015-9; Published 03 May 2017

The financial support for this article was not fully acknowledged. The Acknowledgements should have included the following:

Dr Anne McTiernan was supported in part by a grant from the Breast Cancer Research Foundation.

