# Supplementary material for: Inflammation of mammary adipose tissue occurs in overweight and obese patients exhibiting early-stage breast cancer
Source: NPJ Breast Cancer. 2017 May 3;3:19. doi: 10.1038/s41523-017-0015-9 (PMC5460134; doi:10.1038/s41523-017-0015-9)
Supplement: Supplementary file 1 — Supplementary Table 1 [file 41523_2017_15_MOESM1_ESM.docx]

| **Supplementary Table 1**. Characteristics of the breast cancer patients stratified by presence of CLS (CLS_-postive_-CLS_-negative_); presented as means (SD) or % (n). | | | |  |
| --- | --- | --- | --- | --- |
| *Metabolic parameters* | *CLS negative*  n=*49 ^a^* | *CLS positive*  n=*58 ^a^* | P*-value* |  |
| Cholesterol (mmol/L), mean (SD) | 5.57 (0.99) | 5.65 (0.94) | 0.69 |  |
| HDL-cholesterol (mmol/L), mean (SD) | 1.95 (0.57) | 1.82 (0.52) | 0.20 |  |
| LDL-cholesterol (mmol/L), mean (SD) | 3.37 (0.97) | 3.39 (0.87) | 0.90 |  |
| Triglycerides (mmol/L), mean (SD) | 0.89 (0.44) | 1.21 (0.56) | 0.0021 |  |
| HDL/total cholesterol ratio, mean (SD) | 0.36 (0.12) | 0.33 (0.10) | 0.11 |  |
| Triglycerides/HDL-cholesterol, mean (SD) | 0.53 (0.40) | 0.78 (0.58) | 0.011 |  |
| CRP (mg/L), median (interquartile range) | 0.70 (1.30) | 1.10 (2.70) | 0.27 |  |
| Glucose (mmol/L), mean (SD) | 5.21 (0.62) | 5.61 (0.61) | 0.0012 | |
| HbA1c | 5.34 (0.36) | 5.61 (0.36) | 0.0002 | |
| ^a^ Number of patients may vary due to missing information  Abbreviations: CLS, crown-like structure; HDL, high-density lipoprotein; LDL, low-density lipoprotein; CRP, C-reactive protein; n, cases; SD, standard deviation. | | | |  |
