# Supplementary figures and images for: Inflammation of mammary adipose tissue occurs in overweight and obese patients exhibiting early-stage breast cancer
Source: NPJ Breast Cancer. 2017 May 3;3:19. doi: 10.1038/s41523-017-0015-9 (PMC5460134; doi:10.1038/s41523-017-0015-9)

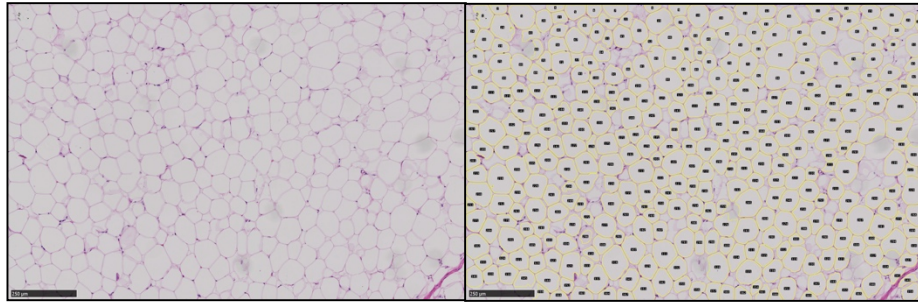

a

A

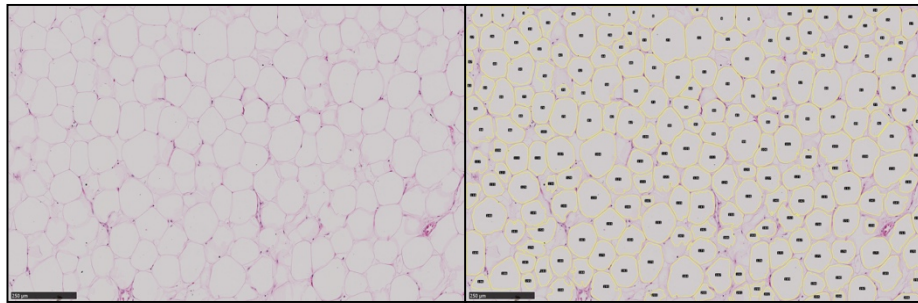

b

B

*Vaysse et al, Supplementary Figure 1*

Supplement: Supplementary file 2 — Supplementary Figure 1 [file 41523_2017_15_MOESM2_ESM.pdf]

a

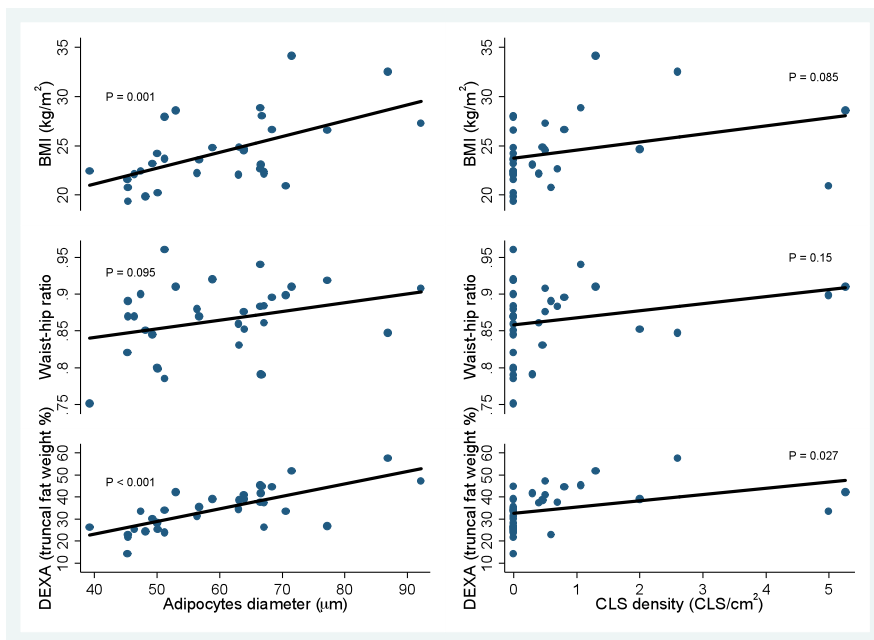

b

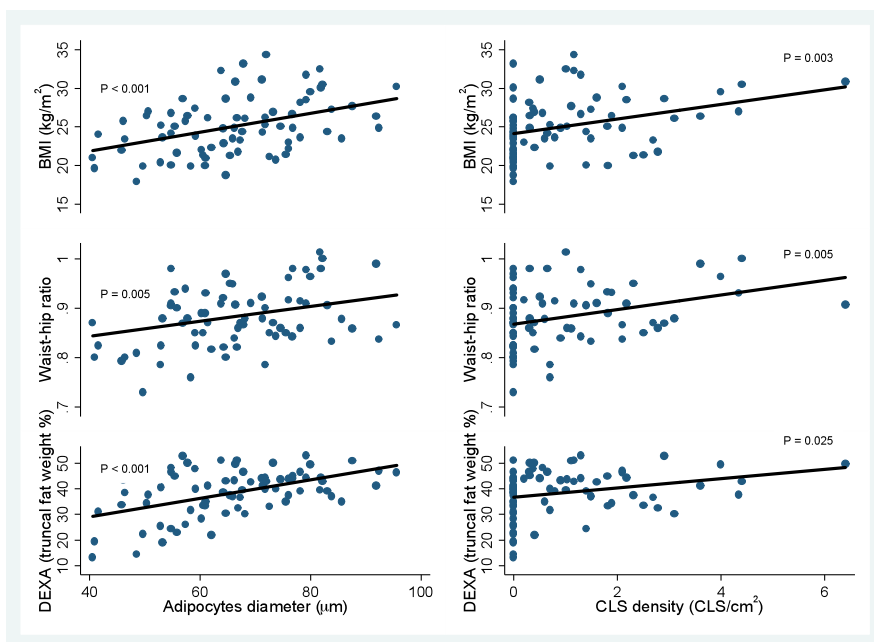

Vaysse et al, Supplementary Figure 2

Supplement: Supplementary file 3 — Supplementary Figure 2 [file 41523_2017_15_MOESM3_ESM.pdf]
